# Sex Differences in Plasma Lysophosphatidic Acid Species in Patients with Alcohol and Cocaine Use Disorders

**DOI:** 10.3390/brainsci12050588

**Published:** 2022-04-30

**Authors:** María Flores-López, Nuria García-Marchena, Pedro Araos, Nerea Requena-Ocaña, Oscar Porras-Perales, Sandra Torres-Galván, Juan Suarez, Nieves Pizarro, Rafael de la Torre, Gabriel Rubio, Juan Jesús Ruiz-Ruiz, Fernando Rodríguez de Fonseca, Antonia Serrano, Francisco Javier Pavón-Morón

**Affiliations:** 1 Instituto de Investigación Biomédica de Málaga—IBIMA, 29590 Málaga, Spain; maria.flores@ibima.eu (M.F.-L.); ngarciam@igtp.cat (N.G.-M.); pedro.araos@ibima.eu (P.A.); nerea.requena@ibima.eu (N.R.-O.); oscarpp84@gmail.com (O.P.-P.); sandra.torres2594@gmail.com (S.T.-G.); juan.suarez@ibima.eu (J.S.); fernando.rodriguez@ibima.eu (F.R.d.F.); javier.pavon@ibima.eu (F.J.P.-M.); 2 Unidad de Gestión Clínica de Salud Mental, Hospital Regional Universitario de Málaga, 29010 Málaga, Spain; 3 Departamento de Psicobiología y Metodología de las Ciencias del Comportamiento, Facultad de Psicología, Universidad de Málaga, 29010 Málaga, Spain; 4 Unidad de Adicciones-Servicio de Medicina Interna, Institut d’Investigació en Ciències de la Salut Germans Trias i Pujol (IGTP), 08916 Badalona, Spain; 5 Facultad de Farmacia, Universidad Complutense de Madrid, 28040 Madrid, Spain; 6 Departamento de Anatomía Humana, Medicina Legal e Historia de la Ciencia, Facultad de Medicina, Universidad de Málaga, 29010 Málaga, Spain; 7 Grup de Recerca en Farmacologia Integrada i Neurociència de Sistemes, Programa de Recerca en Neurociéncia, Institut Hospital del Mar d’Investigacions Mèdiques-IMIM, 08003 Barcelona, Spain; npizarro@imim.es (N.P.); rtorre@imim.es (R.d.l.T.); 8 Centro de Investigación Biomédica en Red de Fisiopatologia de la Obesidad y la Nutrición (CIBEROBN), Instituto de Salud Carlos III, 28029 Madrid, Spain; 9 Servicio de Psiquiatría, Hospital Universitario 12 de Octubre, 28041 Madrid, Spain; gabriel.rubio@salud.madrid.org; 10 Centro Provincial de Drogodependencias de Málaga, Diputación Provincial de Málaga, 29010 Málaga, Spain; jjruiz@malaga.es; 11 Unidad de Gestión Clínica del Corazón, Hospital Universitario Virgen de la Victoria de Málaga, 29010 Málaga, Spain; 12 Centro de Investigación Biomédica en Red de Enfermedades Cardiovasculares (CIBERCV), Instituto de Salud Carlos III, 28029 Madrid, Spain

**Keywords:** lysophosphatidic acid, biomarker, sex, substance use disorder, alcohol, cocaine

## Abstract

Preclinical evidence suggests a main role of lysophosphatidic acid (LPA) signaling in drug addiction. Recently, we reported alterations in the plasma concentrations of LPA species in patients with alcohol use disorder (AUD). As there are sex differences in drug addiction, the main aim of the present study was to investigate whether relevant LPA species (16:0-LPA, 18:0-LPA, 18:1-LPA, 18:2-LPA and 20:4-LPA) were associated with sex and/or substance use disorder (SUD). This exploratory study was conducted in 214 abstinent patients with lifetime SUD, and 91 healthy control subjects. The SUD group was divided according to the diagnosis of AUD and/or cocaine use disorder (CUD). Participants were clinically assessed, and plasma samples were collected to determine LPA species and total LPA. We found that LPA concentrations were significantly affected by sex, and women showed higher concentrations than men. In addition, there were significantly lower 16:0-LPA, 18:2-LPA and total LPA concentrations in patients with SUD than in controls. Namely, patients with CUD and AUD + CUD showed lower LPA concentrations than controls or patients with AUD. In conclusion, our data suggest that LPA species could be potential biomarkers for SUD in women and men, which could contribute to a better stratification of these patients in treatment programs.

## 1. Introduction

Lysophosphatidic acid (LPA, 1-acyl-2-hydroxy-sn-glycero-3-phosphate) is a small endogenous lysophospholipid that is involved in numerous biological processes through specific G protein-coupled receptors (GPCRs) in the CNS and peripheral tissues [1,2]. In mammals, serum and plasma are the major sources of LPA, although this lipid mediator is also found in other biological fluids, such as saliva, tears, semen, and cerebrospinal fluid, at biologically relevant concentrations [3,4].

LPA species are derived from cell membrane phospholipids through different metabolic pathways [1,5], and autotaxin (ATX) is the primary enzyme responsible for the synthesis of LPA [1,4]. These metabolic pathways result in the production of different molecular species of LPA, which vary in the length and saturation degree of the fatty acid moiety [6,7]. The 16:0-LPA, 18:0-LPA, 18:1-LPA, 18:2-LPA and 20:4-LPA species are the most abundant in human blood [8].

LPA species exert their effects through a complex family of cognate GPCRs (i.e., LPA1-6) that are ubiquitously distributed across the body and activate different signal transduction pathways [2]. Through activation of these receptors, LPA plays a key role in many physiological and developmental processes, participating in cell proliferation, migration, differentiation, and survival functions [3,9]. However, LPA also participates in many pathological processes, and a disruption in LPA-mediated signaling can promote many disease states, such as fibrosis, inflammation, hepatic diseases, cardiovascular diseases, and cancer [10,11,12,13,14]. Moreover, alterations in LPA can also result in neurodevelopmental disturbances and neuropsychiatric diseases, such as schizophrenia, Alzheimer’s disease, and autism [15,16]. As the ATX-LPA axis is usually altered under these pathological states, circulating concentrations of ATX and/or LPA species have been proposed as new biomarkers for the detection of relevant diseases, such as different types of cancer [17,18].

A growing body of evidence suggests a potential role of LPA in substance use disorders (SUDs) and common substance-use-related diseases. In this regard, preclinical studies from our group have proposed the involvement of 18:1-LPA and its receptor LPA1 in the modulation of alcohol-related behaviors [19,20] and cocaine addiction processes [21,22]. In addition to these observations in rodents, we have recently linked LPA signaling to alcohol use disorder (AUD) in humans [23,24]. We have reported that abstinent patients with AUD display lower plasma concentrations of LPA species than healthy control subjects, and this decrease is associated with mild cognitive impairment [24]. These findings indicate that LPA could be a useful biomarker for detecting executive impairment in persons diagnosed with AUD. Moreover, we have also shown that patients with lifetime AUD and alcoholic liver disease have higher plasma ATX concentrations than patients without alcoholic liver disease, which suggests that the ATX-LPA axis might be a reliable diagnostic and/or preventive biomarker for alcoholic liver disease [23]. Unlike AUD, plasma concentrations of ATX and LPA species have not been sufficiently explored in patients with cocaine use disorder (CUD) and the potential role of LPA signaling as biomarker for cocaine addiction has yet to be elucidated.

Despite the potential value of LPA as putative biomarker for SUD, the plasma concentration of this lipid family can be influenced for many demographic and anthropometric factors in healthy subjects [25]. Among these factors, sex has a great influence on LPA concentrations and healthy women have higher LPA concentrations than men [25,26].

As both human and animal studies have reported the existence of sex differences in the progression of drug addiction, relapse and response to treatment (for a review, see [27,28,29]), the main purpose of this exploratory study was to examine the plasma concentrations of LPA species in women and men from a cohort of abstinent patients diagnosed with lifetime AUD and/or CUD.

## 2. Materials and Methods

### 2.1. Participants and Recruitment

This exploratory study was performed in abstinent patients from outpatient treatment programs for alcohol or cocaine, and healthy control subjects. The final sample included a total of 305 Caucasian participants, who were first divided into two groups: (i) SUD group, 214 abstinent patients diagnosed with lifetime SUD; and (ii) control group, 91 healthy control subjects. Subsequently, the SUD group was also divided into three subgroups of patients based on the diagnosis of lifetime AUD and/or CUD: the AUD (*n* = 73), CUD (*n* = 48) and AUD + CUD (co-existence of AUD and CUD, *n* = 93) subgroups.

Initially, 229 participants were enrolled in outpatient treatment programs for alcohol and/or cocaine at Hospital Universitario 12 de Octubre (Madrid, Spain), Hospital Regional Universitario de Málaga (Málaga, Spain) and Centro Provincial de Drogodependencias (Málaga, Spain) (multicenter cohort of patients with lifetime SUD from the Spanish network for addictive disorders (Red de Trastornos Adictivos, RETICS, Instituto de Salud Carlos III)). In addition, 100 healthy control subjects were initially recruited from a multidisciplinary staff cohort of volunteers working at the Spanish National Public Health System. The control group had no differences in sex composition, age and body mass index (BMI) relative to the SUD group.

### 2.2. Eligibility Criteria

Participation was voluntary but all participants had to meet the following inclusion criteria: 18 years of age or older (up to 60 years) for all participants and diagnosis of lifetime AUD and/or CUD for the SUD group. The exclusion criteria included a personal health record of chronic inflammatory disorders (except for digestive diseases) and infectious diseases (i.e., HIV, hepatitis B, hepatitis C and COVID-19), cognitive or language limitations precluding evaluation, pregnancy or breastfeeding and current substance use, except for nicotine and caffeine, for all participants. In addition, the exclusion criteria for the control group included a personal health record of psychiatric disorders and a personal history of problematic use of addictive substances.

Fifteen patients and 9 controls were finally excluded based on the eligibility criteria.

### 2.3. Ethics Statements

After hearing about the study, each participant signed a written informed consent form. All participants were given the opportunity to express any questions or concerns. The Regional Ethics Committee gave its approval to the study and the recruitment protocols (i.e., Portal de Ética de la Investigación Biomédica de Andalucía-PEIBA and Hospital Regional Universitario de Málaga (Consejería de Salud y Familias, Junta de Andalucía) (PND2018/033) in agreement with the World Medical Association Declaration of Helsinki (Ethical Principles for Medical Research Involving Human Subjects, 64th WMA General Assembly, Fortaleza, Brazil, 2013) and the General Data Protection Regulation (EU) 2016/679 (GDPR) on data protection and privacy in the European Union (EU) and the European Economic Area (EEA). To guarantee confidentiality and privacy, all data were assigned a code number.

### 2.4. Clinical Evaluations

All individuals were assessed by two distinct psychiatric interviews, depending on the sample group, by skilled and experienced psychologists. For all patients, the Spanish version of the “Psychiatric Research Interview for Substance and Mental Diseases” (PRISM) was typically employed. The PRISM is a semi-structured interview based on the DSM-IV-TR criteria that has shown good to exceptional validity and test–retest reliability in the diagnosis of substance-induced disorders and major mental disorders in the addicted population [30,31]. Variables related to abstinence, severity of SUD (calculated with the addition of criteria for SUD based on the current DSM-5), mental and medical comorbidity, psychotropic medication use were collected for this study. Control individuals were evaluated with the Spanish version of the Composite International Diagnostic Interview (CIDI) for the identification of mental disorders [32] and the PRISM module 1 for sociodemographic and physiological variables [23,33].

### 2.5. Collection and Processing of Plasma Samples

After overnight fasting and before the clinical evaluations, blood samples were obtained by competent nurses in the morning. To collect plasma, venous blood samples were extracted into 10-mL K2 EDTA tubes (BD, Franklin Lakes, NJ, USA) and centrifuged at 2200× *g* for 15 min (4 °C). Individual plasma samples were tested for infectious diseases using rapid tests for HIV, hepatitis B, hepatitis C (Strasbourg, Cedex, France) and SARS-CoV-2 (Bio-Connect, Huissen, The Netherlands). Following laboratory safety standards, infected samples were discarded. Individually recorded plasma samples were kept in aliquots at 80 °C until analysis for LPA species.

### 2.6. Analysis of LPA Species

The LPA species of saturated (i.e., 1-palmitoyl-LPA (16:0-LPA) and 1-stearoyl-LPA (18:0-LPA)) and unsaturated (i.e., 1-oleoyl-LPA (18:1-LPA), 1-linoleoyl-LPA (18:2-LPA) and 1-arachidonoyl-LPA (20:4-LPA)) fatty acids species are the most abundant in human blood [8] and were determined in plasma using an extraction protocol and a liquid chromatography with the tandem mass spectrometry (LC-MS/MS) method, based on a validated, previously reported procedure [24]. Specifically, the detection of LPA species was performed using an ACQUITY UPLC system (Waters Associates, Milford, MA, USA) for chromatographic separation and a Xevo TQ-S micro triple quadrupole mass spectrometer (Waters Associates, Milford, MA, USA) with an orthogonal Z-spray-electrospray interface (ESI). Data management was performed with The TargetLynx XS application/option in the Waters MassLynx Software v4.1. Plasma concentrations of LPA species were expressed as nmol/L and LPA total was calculated by adding the concentrations of the measured LPA species [8,24,34].

A complete description of the protocol for the analysis of LPA species in plasma is shown in the Supplementary Materials (Text S1, Table S1 and Figure S1).

### 2.7. Statistical Analysis

The number and percentage of individuals (*n* (%)), mean ± standard deviation (SD), or median and interquartile range (median (IQR)) were used in the tables. Statistical differences in categorical variables were evaluated with the chi-square test or the Fisher’s exact test, whereas differences in continuous variables were evaluated with the Student’s *t*-test for a normal distribution or the Mann–Whitney U test for a non-normal distribution.

Analysis of covariance (ANCOVA) was used to evaluate the main effects and interaction of primary independent variables (i.e., sample group (the SUD and control groups) and sample subgroup (the AUD, CUD and AUD + CUD subgroups and the control group)) on 16:0-LPA, 18:0-LPA, 18:1-LPA, 18:2-LPA, 20:4-LPA and total LPA concentrations while adjusting for age and BMI. Raw data for LPA concentrations were log10-transformed because their distribution was positively skewed to ensure statistical assumptions of the ANCOVA. The Sidak‘s test was used for post hoc pairwise comparisons between subgroups. The estimated marginal means and 95% confidence interval (95% CI) of log10-transformed LPA concentrations were back-transformed in the figures.

Correlation analyses with the Pearson’s correlation coefficient (r) were performed with log10-transformed LPA concentrations (LPA species and total LPA), age and BMI.

The GraphPad Prism version 5.04 (GraphPad Software, San Diego, CA, USA) and IBM SPSS Statistics version 22 (IBM, Armonk, NY, USA) were used for the statistical studies. A *p*-value of less than 0.05 was considered statistically significant.

## 3. Results

### 3.1. Sociodemographic Characteristics and LPA Concentrations Based on Sample Group

A sociodemographic description of the sample, divided into the SUD (*n* = 214) and control (*n* = 91) groups, is shown in Table 1. As expected, there were no statistical differences between both groups in terms of sex, age and BMI, and the participants were mainly men (83%), with a mean age of 41 years and a mean BMI of 25 kg/m^2^. Marital status and education were also evaluated in both groups but there were only significant differences in education (*p* < 0.001). Thus, patients with SUD showed a lower proportion of tertiary education (16%) and higher primary education (24%) than control subjects (40% and 3%, respectively).

The relative abundance of the LPA species in the plasma samples was similar in both groups (total LPA > 18:2-LPA > 20:4-LPA > 16:0-LPA > 18:1-LPA > 18:0-LPA). Raw data for concentrations of LPA species and total LPA were compared between both the SUD and control groups using a non-parametric test (Table 2). However, there were only significant differences in 18:2-LPA (1-linoleoyl-LPA), where patients with SUD had significantly lower 18:2-LPA levels than the control subjects (*p* < 0.01).

### 3.2. Correlation Analyses between LPA Concentrations and Age and BMI

Although there were no significant differences in age and BMI when both groups were compared, we explored the association between log10-transformed values of LPA and both physiological variables in the sample based on the diagnosis of SUD. As shown in Table 3, the Pearson’s correlation coefficients revealed significant associations between the majority of LPA species and age in all groups of patients. Thus, there were significant positive correlations between 16:0-LPA, 18:1-LPA, 18:2-LPA, 20:4-LPA and total LPA concentrations and age in the total sample and both SUD and control groups. In contrast, log10-transformed LPA concentrations were not correlated with BMI.

### 3.3. LPA Concentrations Based on Sample Group and Sex

Plasma concentrations of LPA species were examined according to the diagnosis of lifetime SUD and sex. For this purpose, raw data for LPA concentrations were log10-transformed to ensure statistical assumptions of the two-way ANCOVA, with sample group and sex as factors, while controlling for age and BMI.

**Figure 1:**
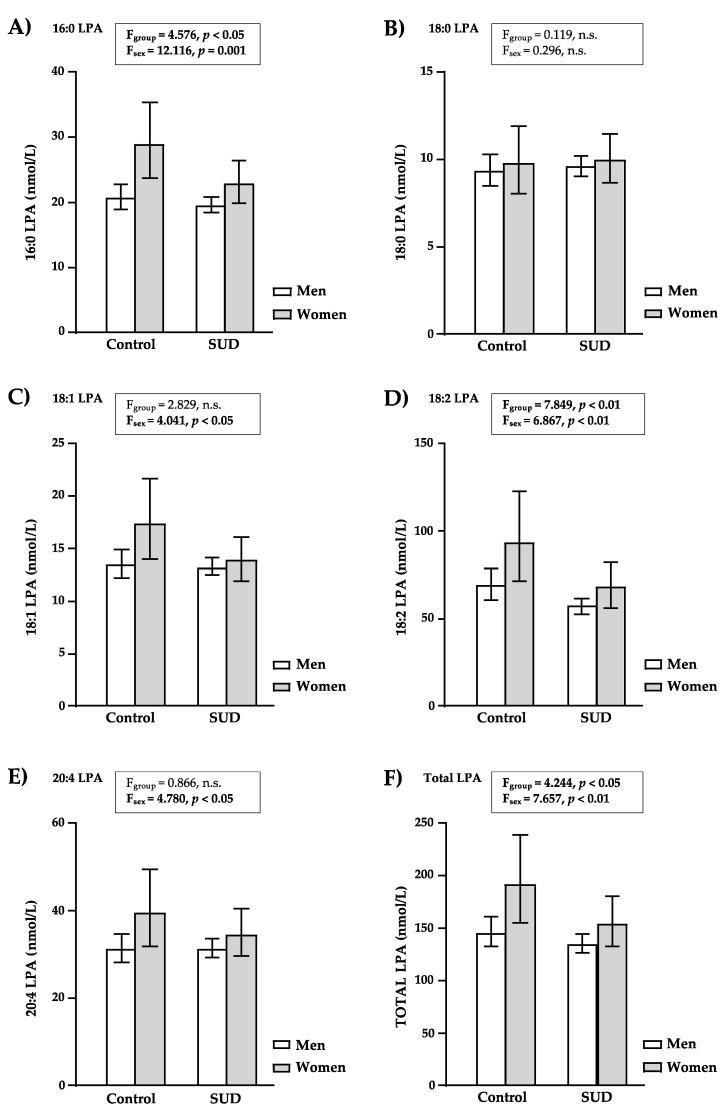
Plasma concentrations of LPA species and total LPA in the sample based on the sample group and sex factors. (**A**) 16:0-LPA concentrations; (**B**) 18:0-LPA concentrations; (**C**) 18:1-LPA concentrations; (**D**) 18:2-LPA concentrations; (**E**) 20:4-LPA concentrations; and (**F**) total LPA concentrations (nmol/L). Log10-transformed concentrations of LPA species and total LPA were analyzed using two-way ANCOVA with sample group (SUD and control) and sex (women and men) as factors while controlling for age and BMI. The bars represent the estimated marginal means and 95% CI after back-transformation. F-statistics and *p*-values of ANCOVA are shown. (n.s.) *p* > 0.05 denotes no significant differences.

Figure 1 shows the back-transformation of the estimated marginal means and 95% CI of the LPA species and total LPA based on diagnosis of SUD and sex. Overall, whereas lower LPA concentrations (16:0-LPA, 18:1-LPA and total LPA) were observed in abstinent patients with SUD, clear sex differences were observed for LPA and women had higher concentrations for the majority of LPA species and total LPA. In all the cases, there were no significant interactions between the sample group and sex factors on plasma concentrations of the distinct LPA species and total LPA.

#### 3.3.1. LPA Concentrations in Controls and Patients with SUD

The analysis revealed a significant main effect of the sample group (control versus SUD group) factor on 16:0-LPA (F_(1, 299)_ = 4.576, *p* = 0.033) (Figure 1A), 18:2-LPA (F_(1, 299)_ = 7.849, *p* = 0.005) (Figure 1C) and total LPA (F_(1, 299)_ = 4.244, *p* = 0.040) (Figure 1E) concentrations. For all three cases, patients diagnosed with SUD had significantly lower levels than controls (16:0-LPA, 21.09 (19.50–22.80) nmol/L and 24.43 (21.83–27.35) nmol/L; 18:2-LPA, 62.37 (56.36–69.02) nmol/L and 80.05 (69.34–92.90) nmol/L; and total LPA, 144.54 (133.35–156.68) nmol/L and 167.49 (149.28–188.36) nmol/L; respectively).

#### 3.3.2. LPA Concentrations in Men and Women

In addition to the sample group factor, there was a significant main effect of the sex factor on 16:0-LPA (F_(1, 299)_ = 12.116, *p* = 0.001) (Figure 1A), 18:1-LPA (F_(1, 299)_ = 4.041, *p* = 0.045) (Figure 1C), 18:2-LPA (F_(1, 299)_ = 6.967, *p* = 0.009) (Figure 1D), 20:4-LPA (F_(1, 299)_ = 4.780, *p* = 0.030) (Figure 1E) and total LPA (F_(1, 299)_ = 7.657, *p* = 0.006) (Figure 1F) concentrations. For all cases, women had significantly higher levels than men (16:0-LPA, 25.70 (22.65–29.11) nmol/L and 20.04 (18.92–21.23) nmol/L; 18:1-LPA, 15.56 (13.58–17.78) nmol/L and 13.37 (12.56–14.19) nmol/L; 18:2-LPA, 79.98 (67.76–94.41) nmol/L and 62.66 (58.08–67.61) nmol/L; 20:4-LPA, 37.07 (32.36–42.56) nmol/L and 31.33 (29.38–33.34) nmol/L; and total LPA, 172.58 (151.36–196.79) nmol/L and 140.60 (132.13–149.28) nmol/L; respectively).

16:0-LPA, 18:2-LPA and total LPA concentrations were significantly affected by both sample group and sex. Therefore, while women with lifetime SUD had the lowest levels, women in the control group had the highest levels. In contrast, 18:0-LPA concentrations showed no significant main effects of both factors.

### 3.4. LPA Concentrations Based on Sample Subgroup and Sex

**Figure 2:**
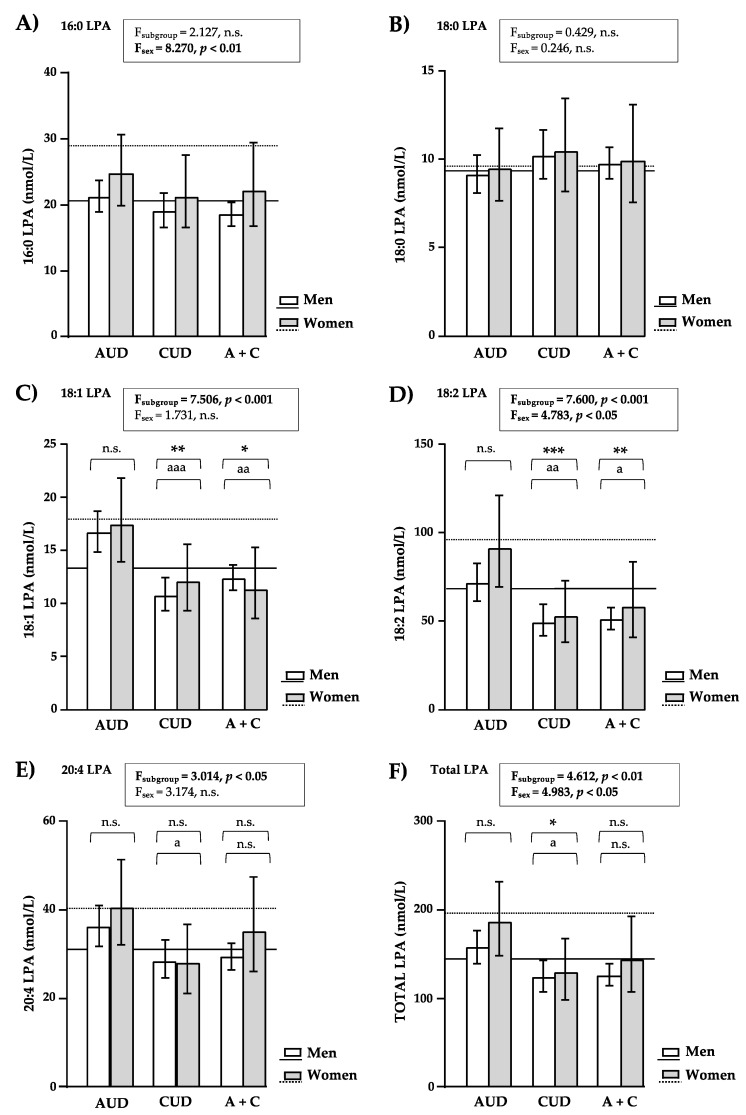
Plasma concentrations of LPA species and total LPA in the sample based on the sample subgroup and sex factors. (**A**) 16:0-LPA concentrations; (**B**) 18:0-LPA concentrations; (**C**) 18:1-LPA concentrations; (**D**) 18:2-LPA concentrations; (**E**) 20:4-LPA concentrations; and (**F**) total LPA concentrations (nmol/L). Log10-transformed concentrations of LPA species and total LPA were analyzed using two-way ANCOVA with sample subgroup (the AUD, CUD, AUD + CUD subgroups and the control group) and sex (women and men) as factors while controlling for age and BMI. The bars represent the estimated marginal means and 95% CI after back-transformation. The horizontal lines represent the estimated marginal means after back-transformation of women (dotted) and men (straight) in the control group. F-statistics and *p*-values of ANCOVA are shown. (*) *p* < 0.05, (**) *p* < 0.01 and (***) *p* < 0.001 denote significant differences compared with the control group. (a) *p* < 0.05, (aa) *p* < 0.01 and (aaa) *p* < 0.001 denote significant differences compared with the AUD subgroup. (n.s.) *p* > 0.05 denotes no significant differences.

As the SUD group was composed of patients diagnosed with AUD and/or CUD, the SUD group was divided into three subgroups: the AUD, CUD and AUD + CUD subgroups. Therefore, we explored log10-transformed concentrations of LPA species in the subgroups of SUD and the control group using a two-way ANCOVA with sample subgroup and sex as factors, while controlling for age and BMI. Figure 2 shows the back-transformation of the estimated marginal means and 95% CI of the LPA species and total LPA.

Despite the division of the SUD group into three subgroups and differences in the sex composition of these subgroups, there were no significant interaction effects between the subgroup and sex factors on LPA concentrations either but there were significant main effects of both factors.

#### 3.4.1. LPA Concentrations in Patients with AUD and/or CUD

Two-way ANCOVA revealed a significant main effect of the sample subgroup factor on 18:1-LPA (F_(3, 295)_ = 7.506, *p* < 0.001) (Figure 2C), 18:2-LPA (F_(3, 295)_ = 7.600, *p* < 0.001) (Figure 2D), 20:4-LPA (F_(3, 295)_ = 3.014, *p* = 0.030) (Figure 2E) and total LPA (F_(3, 295)_ = 4.612, *p* = 0.004) (Figure 2F) concentrations. The post hoc pairwise comparisons showed differences among the subgroups of SUD and the control group. Thus, patients with AUD had no significant differences in the LPA concentrations compared with control subjects. In contrast, patients with CUD had significantly lower levels of 18:1-LPA (*p* < 0.01), 18:2-LPA (*p* < 0.001) and total LPA (*p* < 0.05) than control subjects; and patients with AUD + CUD had significantly lower levels of 18:1-LPA (*p* < 0.05) and 18:2-LPA (*p* < 0.01). In addition, comparisons among the subgroups of SUD showed that while patients with CUD had significantly lower levels of 18:1-LPA (*p* < 0.001), 18:2-LPA (*p* < 0.01), 20:4-LPA (*p* < 0.05) and total LPA (*p* < 0.05) than patients with AUD, patients with AUD + CUD had significantly lower levels of 18:1-LPA (*p* < 0.01) and 18:2-LPA (*p* < 0.05) than patients with AUD. No differences were found between patients with CUD and patients with AUD + CUD, which suggests that the presence of cocaine is the main factor for the reduction in certain LPA species.

#### 3.4.2. LPA Concentrations in Men and Women of the Subgroups of Patients with SUD

Regarding sex, there was a significant main effect of the sex factor on 16:0-LPA (F_(1, 295)_ = 8.270, *p* = 0.004) (Figure 2A), 18:2-LPA (F_(1, 295)_ = 4.783, *p* = 0.030) (Figure 2D) and total LPA (F_(1, 295)_ = 4.983, *p* = 0.026) (Figure 2F) concentrations. As expected, women had significantly higher levels of 16:0-LPA, 18:2-LPA and total LPA concentrations than men.

18:2-LPA and total LPA concentrations were significantly affected by both sample subgroup and sex. In this case, we cannot state that men with lifetime CUD had the lowest levels because we observed no differences between men and women in this subgroup.

### 3.5. Clinical and Psychiatric Variables of the Subgroups of SUD

The clinical characterization of abstinent patients with SUD was based on the type of SUD (AUD, CUD and AUD + CUD subgroups) and sex. We assessed relevant clinical variables such as abstinence duration, DSM criteria for AUD and/or CUD, comorbid SUDs, comorbid psychiatric disorders, psychiatric medication in the last year and comorbid digestive diseases (mainly alcohol liver disease).

#### 3.5.1. Characterization Based on the Type of SUD

As shown in Table 4, the analysis of subgroups of SUD was limited by the underrepresentation of women with respect to men in all the subgroups (23–10%). This is a realistic picture of the current outpatient treatment programs for alcohol and/or cocaine, where the presence of women does not reflect the real prevalence of the SUD problem. Thus, we performed a global analysis of clinical data, disregarding the sex variable for the subgroups, although we performed the analysis for sex differences in the overall SUD group (see below). Following these premises, we found significant differences in the prevalence of comorbid digestive diseases (*p* < 0.001) because patients with AUD had higher cases of digestive diseases than patients with CUD and patients with AUD + CUD (33%, 2% and 7%, respectively). Although no significant differences were found, patients with AUD + CUD showed a higher prevalence of comorbid SUDs (34%, mainly associated with cannabis and sedatives) and comorbid psychiatric disorders (67%, mainly mood, anxiety and personality disorders) than other subgroups.

#### 3.5.2. Characterization Based on Sex

To analyze the influence of sex in global clinical characteristics of recruited patients, the SUD group was also divided into women and men (Table 5). There were significant differences in the prevalence of comorbid psychiatric disorders (*p* < 0.05), psychotropic medication use (*p* < 0.05) and comorbid digestive diseases (*p* < 0.05). Overall, women with lifetime SUD showed a lower prevalence of comorbid SUDs (14%), but a significantly higher prevalence of comorbid psychiatric disorders (77%, mainly anxiety) than men. Therefore, women with SUD had a significantly higher use of psychotropic medication than men (91% and 64%), respectively). Finally, the incidence of digestive diseases was significantly lower in women than in men with SUD (3% and 16%, respectively).

This clinical and psychiatric characterization of patients with SUD suggests a worse clinical condition in those with the coexistence of AUD and CUD (except for comorbid digestive diseases) and in women because of the high prevalence of psychiatric comorbidity.

## 4. Discussion

The identification of plasma biomarkers for SUD and comorbid diseases can be relevant in the stratification and treatment of patients. As there are sex differences in the progression of drug addiction and in the response to treatment, it is important to evaluate the influence of sex on these putative biomarkers. In this exploratory study, plasma concentration of different species of LPA (16:0-LPA, 18:0-LPA, 18:1-LPA, 18:2-LPA and 20:4-LPA) and total LPA were examined in men and women from a cohort of patients with SUD. As preclinical studies have reported an influence of LPA on alcohol and cocaine addiction [19,20,21,22], we also examined the plasma concentration of LPA species in these SUD patients according to the substance use (alcohol (AUD) and/or cocaine (CUD)). As expected, men with SUD seeking treatment were more common than women (83% and 16%, respectively) but the clinical characterization revealed that women showed a higher prevalence of comorbid psychiatric disorders (mainly anxiety disorders) than men (77% and 56%, respectively). The relative abundance of the LPA species in the plasma of the total sample (18:2-LPA > 20:4-LPA > 16:0-LPA > 18:1-LPA > 18:0-LPA) was similar to previous reports in humans [34].

The main findings of the present study are summarized as follows: (1) In the total sample, there were sex differences on LPA concentrations and women displayed higher concentrations than men. (2) There were significant positive correlations between 16:0-LPA, 18:1-LPA, 18:2-LPA, 20:4-LPA or total LPA concentrations and age in the SUD and control groups, but no correlations were found with BMI. (3) Patients with SUD displayed lower 16:0-LPA, 18:2-LPA and total LPA concentrations than healthy controls. (4) 16:0-LPA, 18:2-LPA and total LPA concentrations were affected by diagnosis of SUD and sex, although there were no interactions between both factors. (5) Finally, the different species of LPA and total LPA concentrations were affected by the type of SUD. Thus, patients with CUD and AUD + CUD displayed lower LPA concentrations than control or AUD groups.

Previous studies in healthy controls have reported higher LPA concentrations in women than in men [25,26]. Similarly, we have also described higher plasma LPA concentrations in women with AUD than in men [24]. In agreement with these previous reports, the present results showed that the plasma concentration of some species of LPA and total LPA was affected by sex, showing higher concentrations in women than in men. We observed these differences in both groups, SUD and control, which suggests that the influence of sex on LPA concentrations was not dependent on drug consumption. As mentioned previously, men with SUD seeking treatment were more common than women. In fact, the difficulty in the recruitment of female patients with SUD is a main limitation in studies according to sex. Women have been underestimated in most of the studies related to substance use, which is a reflection of the significant barriers (e.g., structural, social, cultural, personal and physiological factors) that women encounter in accessing substance use treatment services [35]. In this regard, several studies have reported that women have lower rates of cocaine use and attend treatment programs to a lesser extent than men [36,37,38]. In agreement with that, in our study, we found a lower rate of women than men in the outpatient treatment programs for alcohol and cocaine. Moreover, we observed in our cohort that women seek treatment for substances when their SUD is more severe and/or when there are comorbid psychiatric disorders that aggravate their SUD. We also observed a greater consumption of psychiatric medication in women than in men. All these considerations are important when exploring the phenotyping of women with SUD. Therefore, the present data showing sex differences in LPA concentrations might also be associated with specific sociodemographic and clinical characteristics of the female SUD population.

Another finding of this study was the positive association between plasma concentrations of LPA species or total LPA and age in both control and SUD groups. In contrast, we found no correlations between LPA concentrations and BMI. A previous study in healthy subjects described positive associations between LPA and age and BMI [25]; however, inconsistent findings regarding the influence of age on LPA have also been reported [26,39]. Therefore, two-way ANCOVA was used in our study to control the possible influence of these physiological variables (age and BMI) on LPA concentrations.

The present results showed significantly lower total LPA concentrations in participants with SUD than in control subjects, which is consistent with recent clinical studies from our group [23,24]. Moreover, we also observed significantly lower concentrations of 16:0-LPA and 18:2-LPA in patients with SUD than in control subjects. Although the origin of these changes is unknown, it is possible that circulating fatty acids levels might be affected by alcohol and/or cocaine exposure and, consequently, the production of fatty acid derivatives. Moreover, linoleic acid (18:2-LPA is derived from this fatty acid) is an essential fatty acid; therefore, the effect of diet on LPA species cannot be discarded.

As previously discussed, the existence of a sexual dimorphism in the plasma expression of LPA species and total LPA was observed in both control and SUD groups. Although there were no significant interactions between sample group and sex factors, we observed that women with SUD had the lowest plasma concentrations of 16:0-LPA, 18:0-LPA and total LPA, whereas control women displayed the highest concentrations of these lipids. It is known that LPA plays a key role in the control of emotional behaviors [40], and its receptor LPA1 is involved in anxious depression [41]. Thus, the existence of these sex differences in plasma LPA concentrations might be associated with the high prevalence of anxiety disorders in women diagnosed with SUD.

As the SUD group was composed of patients diagnosed with AUD and/or CUD, we also analyzed the plasma concentrations of LPA species in the subgroups of SUD (AUD, CUD and AUD + CUD) to evaluate their association with different substance uses (alcohol and/or cocaine). As expected, the AUD subgroup showed a higher prevalence of comorbid digestive diseases than the other subgroups, and the AUD + CUD subgroup showed a higher prevalence of comorbid SUDs and comorbid psychiatric disorders than the other subgroups. When we evaluated the plasma concentrations of the different species of LPA and total LPA in these subgroups, we found significantly lower concentrations in patients with CUD or AUD + CUD than in patients with AUD and healthy controls. These results suggest that the main effects observed on LPA concentrations were mainly produced by a pathological use of cocaine, alone or in combination with alcohol. In this regard, numerous preclinical studies have described that the consumption of psychostimulants, such as cocaine, has an impact on LPA/LPA1 pathway [21,22,42]. Thus, monitoring LPA concentrations in the plasma might be a good indicator of psychostimulant abuse in patients with SUD.

## 5. Limitations and Conclusions

Although our findings support the importance of the characterization of plasma LPA concentrations in the context of a history of SUD by accounting for sex, we are aware of the limitations of this exploratory cross-sectional study: (1) There were important statistical limitations related to the sample size of the subgroups of SUD and the limited number of women. Large samples of women should be included to confirm the sexual dimorphism observed in this study; (2) Some sociodemographic differences were associated with the sources of recruitment for the control and SUD groups. We found significant differences in educational levels between both groups, which could be associated with the appearance and severity of SUD [43]; (3) The recruitment of the sample was conducted from outpatient treatment programs and there are some sociodemographic and clinical variables that remain unknown and could affect the validity of our results (e.g., diet and nutrition, income or economic status, non-psychotropic medication, physical activity); (4) The present data were obtained from patients with a history of SUD in abstinence, and additional experimental groups (e.g., active alcohol and cocaine users) should be included; (5) Longitudinal studies are also needed to monitor changes in LPA concentrations during abstinence at different times in the same abstinent SUD patients; (6) Although we have examined the most abundant LPA species in the human blood, we cannot ignore the presence of other acyl-LPA that were not measured, such as 22:6-LPA, whose relative activity could be relevant; and (7) As we did not measure the expression of precursors (e.g., lysophospholipids and glycerol 3-phosphate (G3P)) and metabolic enzymes involved in the metabolism of LPA (e.g., ATX and lipid phosphate phosphatases (LPP_1-3_)), future research is needed to explore these molecules and elucidate potential differences between healthy controls and patients with SUD.

In conclusion, our results suggest that LPA species are altered in patients diagnosed with SUD when compared with healthy controls. Moreover, we also found a sexual dimorphism in plasma LPA concentrations in both control and SUD groups. Further investigation is needed to explore the role of LPA as potential biomarker for SUD, which could contribute to the better stratification of these patients in treatment programs.

## Figures and Tables

**Table 1:** Baseline sociodemographic characteristics.

VARIABLE		GROUP		*p*-Value
		CONTROL	SUD	
		*n* = 91	*n* = 214	
**Sex** **(n (%))**	Women Men	17 (18.7) 74 (81.7)	35 (16.4) 179 (83.6)	0.621 ^a^
**Age** **(years)**	Mean ± SD	40.9 ± 7.0	41.1 ± 9.8	0.861 ^b^
	Median (IQR)	38.0 (37.0–44.0)	40.0 (33.0–49.3)	
**BMI** **(kg/m^2^)**	Mean ± SD	25.5 ± 3.6	25.6 ± 4.1	0.887 ^a^
	Median (IQR)	24.7 (23.3–28.7)	25.2 (22.8–28.0)	
**Marital status** **(n (%))**	Single Married/cohabiting Divorced/separated Widowed	22 (24.2) 50 (54.9) 19 (20.9) 0 (0.0)	82 (38.3) 68 (31.8) 62 (29.0) 2 (0.9)	0.081 ^a^
**Education** **(n (%))**	≤Primary Secondary Tertiary	3 (3.3) 52 (57.1) 36 (39.6)	52 (24.3) 127 (59.3) 35 (16.4)	<0.001 ^a^

^a^*p*-value from the chi-square test; ^b^ *p*-value from the Mann–Whitney U test. *p*-value in bold indicates a statistically significant difference. Abbreviations: BMI = body mass index; IQR = interquartile range; SD = standard deviation; SUD = substance use disorder.

**Table 2:** Plasma concentrations of LPA species and total LPA.

LPA		GROUP		*p*-Value
		CONTROL	SUD	
		*n* = 91	*n* = 214	
**16:0-LPA** **(1-palmitoyl-LPA)** **(nmol/L)**	Median (IQR)	20.72 (16.53–28.49)	19.60 (15.98–25.80)	0.128 ^a^
**18:0-LPA (1-stearoyl-LPA)** **(nmol/L)**	Median (IQR)	9.20 (7.92–10.30)	9.20 (7.86–10.13)	0.867 ^a^
**18:1-LPA** **(1-oleoyl-LPA)** **(nmol/L)**	Median (IQR)	13.70 (9.48–20.20)	13.31 (9.27–17.63)	0.198 ^a^
**18:2-LPA** **(1-linoleoyl-LPA)** **(nmol/L)**	Median (IQR)	70.72 (50.96–104.84)	58.27 (43.38–87.37)	0.004 ^a^
**20:4-LPA** **(1-arachidonoyl-LPA)** **(nmol/L)**	Median (IQR)	30.08 (22.17–47.70)	32.05 (23.89–41.90)	0.969 ^a^
**Total LPA** **(nmol/L)**	Median (IQR)	145.30 (112.80–211.70)	137.52 (106.43–177.39)	0.097 ^a^

^a^ *p*-value from the Mann–Whitney U test. *p*-value in bold indicates a statistically significant difference. Abbreviations: IQR = interquartile range; LPA = lysophosphatidic acid; SUD = substance use disorder.

**Table 3:** Correlation analyses between LPA species and total LPA with age and BMI.

VARIABLES		Age (Years)			BMI (kg/m^2^)		
		TOTAL	CONTROL	SUD	TOTAL	CONTROL	SUD
**16:0-LPA** **(1-palmitoyl-LPA)** **(nmol/L)**	r	+0.177	+0.262	+0.158	+0.045	−0.108	+0.086
	*p*-value	0.002	0.012	0.021	0.435	0.310	0.210
**18:0-LPA (1-stearoyl-LPA)** **(nmol/L)**	r	−0.003	+0.160	−0.033	+0.105	+0.062	+0.117
	*p*-value	0.955	0.130	0.626	0.066	0.559	0.088
**18:1-LPA** **(1-oleoyl-LPA)** **(nmol/L)**	r	+0.414	+0.412	+0.418	+0.027	−0.060	+0.054
	*p*-value	<0.001	<0.001	<0.001	0.645	0.573	0.435
**18:2-LPA** **(1-linoleoyl-LPA)** **(nmol/L)**	r	+0.245	+0.267	+0.240	+0.004	−0.191	+0.055
	*p*-value	<0.001	0.010	<0.001	0.940	0.070	0.425
**20:4-LPA** **(1-arachidonoyl-LPA)** **(nmol/L)**	r	+0.278	+0.345	+0.265	−0.004	−0.014	−0.001
	*p*-value	<0.001	0.001	<0.001	0.946	0.894	0.991
**Total LPA** **(nmol/L)**	r	+0.266	+0.313	+0.255	+0.018	−0.125	+0.060
	*p*-value	<0.001	0.003	<0.001	0.753	0.236	0.214

Correlation analyses were performed after the logarithmic transformation of raw data using the Pearson’s correlation coefficient. *p*-value in bold indicates a statistically significant correlation. Abbreviations: BMI = body mass index; LPA = lysophosphatidic acid; SUD = substance use disorder.

**Table 4:** Clinical and psychiatric variables of the SUD group based on subgroups of SUD.

VARIABLE		SUBGROUP			*p*-Value
		AUD	CUD	AUD + CUD	
		*n* = 73	*n* = 48	*n* = 93	
**Sex** **(n (%)**	Men Women	58 (79.5) 15 (20.5)	37 (77.1) 11 (22.9)	84 (90.3) 9 (9.7)	0.065 ^a^
**Abstinence duration (day)** **(median (IQR))**	Alcohol	90 (45–180)	-	50 (8–150)	0.053 ^b^
	Cocaine	-	15 (4–60)	21 (7–60)	0.221 ^b^
**DSM criteria for SUD** **(median (IQR))**	(0–11) AUD	7.0 (6.0–9.0)	-	8.0 (6.0–9.0)	0.495 ^b^
	(0–11) CUD	-	8.0 (6.3–10.0)	8.0 (6.0–10.0)	0.350 ^b^
**Comorbid SUDs** **(n (%))**	No Yes *	59 (80.8) 14 (19.2)	38 (79.2) 10 (20.8)	61 (65.6) 32 (34.4)	0.054 ^a^
	Cannabis Sedatives Stimulants Opioids	11 (15.1) 2 (2.7) 2 (2.7) 2 (2.7)	6 (12.5) 3 (6.3) 1 (2.1) 1 (2.1)	22 (23.7) 12 (12.9) 1 (1.1) 5 (5.4)	
**Comorbid psychiatric disorders** **(n (%))**	No Yes	32 (43.8) 41 (56.2)	24 (50.0) 24 (50.0)	31 (33.3) 62 (66.7)	0.128 ^a^
	Mood Psychotic Anxiety Eating Personality	30 (41.1) 3 (4.1) 24 (32.9) 2 (2.7) 9 (12.3)	12 (25.0) 2 (4.2) 12 (25.0) 1 (2.1) 14 (29.2)	32 (34.4) 10 (10.8) 25 (26.9) 5 (5.4) 28 (30.1)	
**Psychotropic medication (last year)** **(n (%))**	No Yes	22 (30.1) 51 (69.9)	20 (41.7) 28 (58.3)	26 (28.0) 67 (72.0)	0.237 ^a^
	Antidepressant Anxiolytic Anticraving Antipsychotic	30 (41.1) 26 (35.6) 20 (27.4) 3 (4.1)	14 (29.2) 21 (43.8) 4 (8.3) 4 (8.3)	39 (41.9) 39 (41.9) 18 (19.4) 11 (11.8)	
**Comorbid digestive diseases** **(n (%))**	No Yes	49 (67.1) 24 (32.9)	47 (97.9) 1 (2.1)	87 (93.5) 6 (6.5)	<0.001 ^a^

^a^*p*-value from the Chi-square test or the Fisher’s exact test; ^b^ *p*-value from the Mann–Whitney U test. *p*-value in bold indicates a statistically significant difference. Abbreviations: IQR = interquartile range; SD = standard deviation; SUD = substance use disorder. (*) Comorbid SUD for the AUD + CUD subgroup is considered the diagnosis of other lifetime SUDs different from AUD and CUD.

**Table 5:** Clinical and psychiatric variables of the SUD group based on sex.

VARIABLE		SEX		*p*-Value
		Men	Women	
		*n* = 179	*n* = 35	
**SUD** **(n (%))**	AUD CUD AUD + CUD	58 (32.4) 37 (20.7) 84 (46.9)	15 (42.9) 11 (31.4) 9 (25.7)	0.065 ^a^
**Abstinence duration (day)** **(median (IQR))**	Alcohol	60 (22–150)	90 (30–225)	0.401 ^b^
	Cocaine	18 (4–60)	38 (6–139)	0.114 ^b^
**DSM criteria for SUD** **(median (IQR))**	(0–11) AUD	7.0 (6.0–9.0)	8.0 (7.0–10.0)	0.100 ^b^
	(0–11) CUD	8.0 (6.0–10.0)	8.5 (7.0–10.0)	0.327 ^b^
**Comorbid SUDs** **(n (%))**	No Yes	128 (71.5) 51 (28.5)	30 (85.7) 5 (14.3)	0.094 ^a^
	Cannabis Sedatives Stimulants Opioids	38 (21.2) 15 (8.4) 3 (1.7) 8 (4.5)	2 (5.7) 2 (5.7) 1 (2.9) 0 (0.0)	
**Comorbid psychiatric disorders** **(n (%))**	No Yes	79 (44.1) 100 (55.9)	8 (22.9) 27 (77.1)	0.023 ^a^
	Mood Psychotic Anxiety Eating Personality	61 (34.1) 12 (6.7) 42 (23.5) 1 (0.6) 39 (21.8)	13 (37.1) 3 (8.6) 19 (54.3) 7 (20.0) 12 (34.3)	
**Psychotropic medication (last year)** **(n (%))**	No Yes	65 (36.3) 114 (63.7)	3 (8.6) 32 (91.4)	0.012 ^a^
	Antidepressant Anxiolytic Anticraving Antipsychotic	66 (36.9) 65 (36.3) 33 (18.4) 14 (7.8)	19 (54.3) 21 (60.0) 11 (31.4) 4 (11.4)	
**Comorbid digestive diseases** **(n (%))**	No Yes	150 (83.8) 29 (16.2)	34 (97.1) 1 (2.9)	0.035 ^a^

^a^*p*-value from the Chi-square test or the Fisher’s exact test; ^b^ *p*-value from the Mann–Whitney U test. *p*-value in bold indicates a statistically significant difference. Abbreviations: IQR = interquartile range; SD = standard deviation; SUD = substance use disorder.

## Data Availability

The data presented in this study are available on request from the corresponding author. The data are not publicly available due to ethical and privacy restrictions.

## Supplementary Materials

The following supporting information can be downloaded at: https://www.mdpi.com/article/10.3390/brainsci12050588/s1. Protocol for analysis of LPA species in plasma (Text S1); analytes and SRM conditions for the detection of LPA species in human plasma (Table S1); representative LC-MS/MS chromatogram of a calibration sample containing 25 nmol/L of each analyte (from calibration curve) (Figure S1). Text S1: Protocol for analysis of LPA; Table S1: Analytes and SRM conditions; and Figure S1: LC-MS/MS chromatogram from calibration curve.
