# Supplementary material for: Sex Differences in Plasma Lysophosphatidic Acid Species in Patients with Alcohol and Cocaine Use Disorders
Source: Brain Sci. 2022 Apr 30;12(5):588. doi: 10.3390/brainsci12050588 (PMC9139721; doi:10.3390/brainsci12050588)
Supplement: Supplementary file 1 [file brainsci-12-00588-s001.zip › brainsci-1670042-supplementary.pdf]

SUPPLEMENTARY DATA

1. Text S1. Protocol for analysis of LPA species in plasma

The LPA species of palmitic acid (16:0-LPA), stearic acid (18:0-LPA), oleic acid (18:1-LPA), linoleic acid (18:2-LPA) and arachidonic acid (20:4-LPA) were determined using an extraction protocol followed by Liquid Chromatography with tandem Mass Spectrometry (LC-MS/MS) separation and quantification.

Briefly, 0.2 mL of plasma were spiked with 100 ng of a methanolic solution of 17:0-LPA as internal standard (IS). A liquid–liquid extraction was performed after the addition of 200 μL of butanol. The organic phase was evaporated and reconstituted in 100 μL of mobile phase mixture (80% phase A and 20% phase B, see below) prior to analysis. Stock solutions (100 μg/mL) for each analyte were independently prepared by diluting adequate amounts of standards in methanol, and the working solutions were prepared by mixture of the stock solutions and dilution in methanol.

The calibration curves for all the target analytes contained the following concentrations: 0.2, 0.5, 1.0, 1.5, 2.0, 4.0, 6.0, 8.0, 10.0 μg/mL; and their linearity was verified with a coefficient of determination (R2) higher than 0.99 in all cases. Before the quantification of samples and in order to verify matrix effect and recovery of the analytical method for each analyte, the calibration curves were prepared in both plasma and water. In all cases, matrix effects lower than 6% and recoveries higher than 66% were achieved. At this point, the calibration curves to perform quantification of samples were prepared in water by duplicate in each analytical batch. The procedure of lipid analysis in plasma was performed by a validated method previously described in clinical samples [1]. Quantification of LPA species in plasma was performed using an ACQUITY UPLC system (Waters Associates, Milford, MA, USA) for the chromatographic separation coupled to a triple quadrupole (Xevo TQ-S micro) mass spectrometer provided with an orthogonal Z-spray-electrospray interface (ESI) (Waters Associates, Milford, MA, USA). The drying and nebulizing gas was nitrogen. The desolvation gas flow was set to 1,200 L/h and the cone gas flow to 50 L/h. A capillary voltage of 3 kV was used in negative ionization mode. The nitrogen desolvation temperature was set to 600 °C and the source temperature to 150 °C. Collision gas was argon and the injection volume was 5 μL. The chromatographic separation was achieved at 30 °C using an ACQUITY UPLC BEH C18 column (2.1 × 100 mm × 1.7 μm) (Waters Associates, Milford, MA, USA), at a flow rate of 300 μL/min. Mobile phase A was ammonium formate 1 mM with formic acid (0.01%, v/v) dissolved in methanol. Mobile phase B was ammonium formate 1 mM with formic acid (0.01%, v/v) in water. A gradient program was employed for the separation of the analytes; the percentage of mobile phase B linearly changed as follows: 0 min, 20%; 0.2 min, 20%; 6 min, 10%; 6.5 min, 10%; 7 min, 20%; 8 min, 20%. Total run time was 8 min. Analytes were determined by a Selected Reaction Monitoring (SRM) method by acquiring two transitions for each compound as specified (Table S1). The most specific transition was selected for quantitative purposes. MassLynx software V4.1 and TargetLynx XS were used for data management. Finally, the LPA species plasma concentrations were recalculated to molar concentration (nmol/L).

2. Table S1. Analytes and SRM conditions for the detection of LPA species in human plasma

Analyte	Precursor (m/*z*)	Product (m/*z*)	Collision energy (eV)
**16:0 LPA**	409	79	40
		153	20
**17:0 LPA***	423	79	50
		153	20
**18:0 LPA**	437	79	40
		153	20
**18:1 LPA**	435	79	50
		153	30
**18:2 LPA**	433	79	50
		153	20
**20:4 LPA**	457	79	40
		153	20

(*) IS

3. Figure S1. Representative LC-MS/MS chromatogram of a calibration sample containing 25 nmol/L of each analyte (from calibration curve)

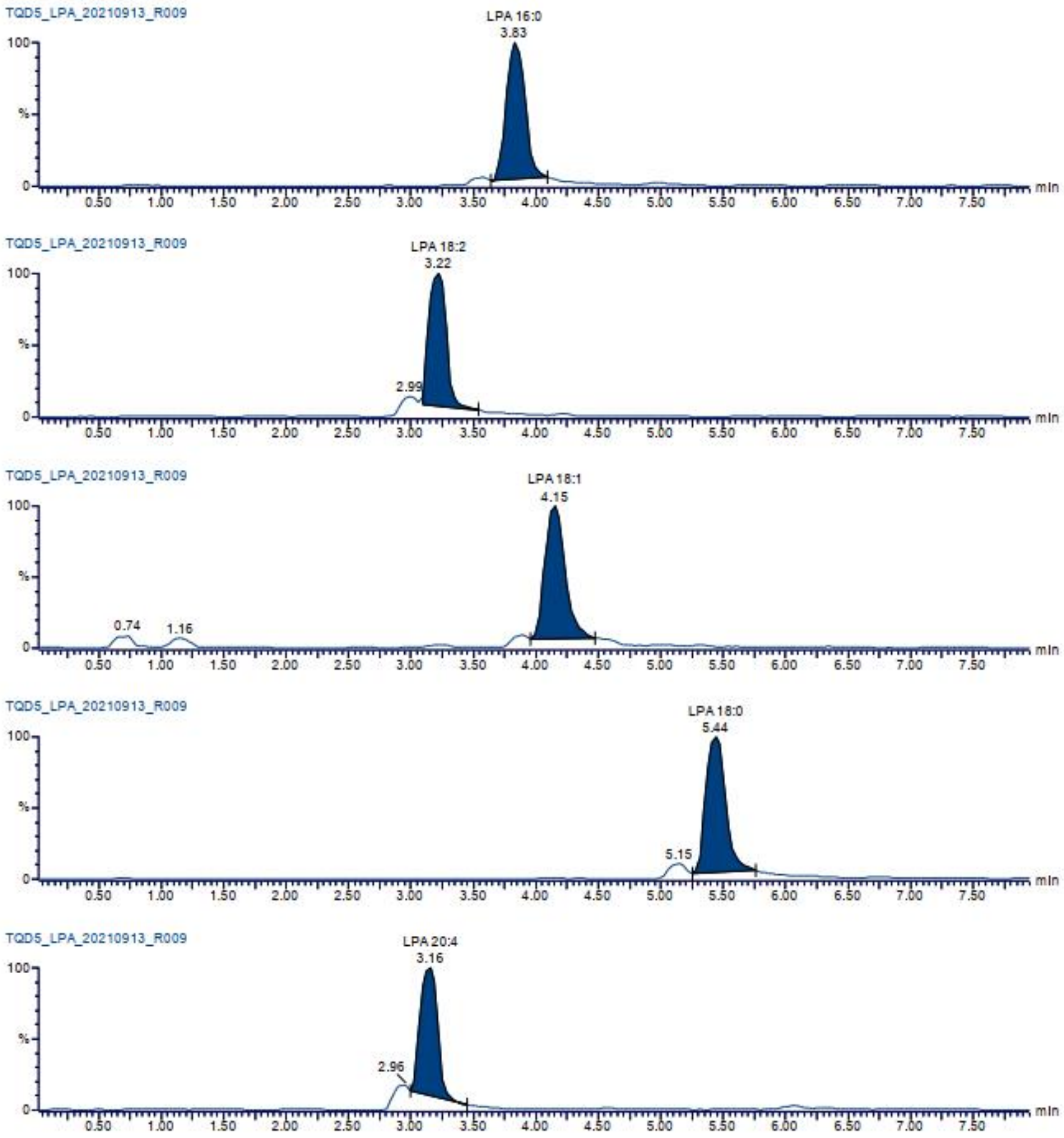

References

1. Garcia-Marchena, N.; Pavon, F.J.; Pastor, A.; Araos, P.; Pedraz, M.; Romero-Sanchiz, P.; Calado, M.; Suarez, J.; Castilla-Ortega, E.; Orio, L.; et al. Plasma concentrations of oleoylethanolamide and other acylethanolamides are altered in alcohol-dependent patients: effect of length of abstinence. *Addict. Biol.* **2017**, *22*, 1366-1377.
